# Methyl pyrazine-2-carboxyl­ate

**DOI:** 10.1107/S1600536809043451

**Published:** 2009-10-28

**Authors:** Chang-Hua Zhang, Xue-Jie Tan, Dian-Xiang Xing

**Affiliations:** a Department of Chemistry and Chemical Industry, Binzhou University, Binzhou, Shandong Province 256600, People’s Republic of China; b Department of Chemical Industry, Shandong Institute of Light Industry, Jinan 250353, People’s Republic of China

## Abstract

The title compound, C_6_H_6_N_2_O_2_,  is approximately planar [r.m.s. deviation = 0.0488 (3) Å]. In the crystal, weak inter­molecular C—H⋯O and C—H⋯N inter­actions join the mol­ecules into an infinite three-dimensional network.

## Related literature

For the synthetic procedure, see: Kim *et al.* (2004 ▶). For reduction of heteroaromatic esters, see: Boechat *et al.* (2005 ▶). For a description of weak hydrogen bonds, see: Desiraju & Steiner (1999 ▶).
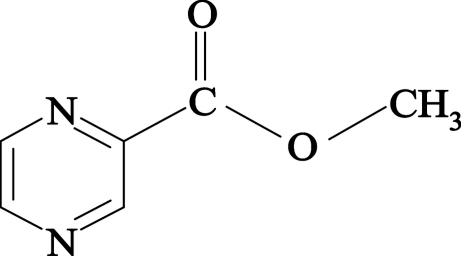


## Experimental

### 

#### Crystal data

C_6_H_6_N_2_O_2_
*M* *_r_* = 138.13
Orthorhombic, 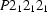

*a* = 3.865 (2) Å
*b* = 6.690 (4) Å
*c* = 24.92 (2) Å
*V* = 644.4 (7) Å^3^
*Z* = 4
Mo *K*α radiation
μ = 0.11 mm^−1^
*T* = 298 K
0.32 × 0.12 × 0.05 mm

#### Data collection

Bruker SMART CCD area-detector diffractometer
Absorption correction: multi-scan (*SADABS*; Bruker, 2000 ▶) *T*_min_ = 0.980, *T*_max_ = 0.994
3378 measured reflections
757 independent reflections
505 reflections with *I* > 2σ(*I*)
*R*_int_ = 0.080

#### Refinement

*R*[*F* ^2^ > 2σ(*F* ^2^)] = 0.066
*wR*(*F* ^2^) = 0.153
*S* = 1.05
757 reflections
91 parameters
H-atom parameters constrained
Δρ_max_ = 0.18 e Å^−3^
Δρ_min_ = −0.17 e Å^−3^

### 

Data collection: *SMART* (Bruker, 2000 ▶); cell refinement: *SAINT* (Bruker, 2000 ▶); data reduction: *SAINT*; program(s) used to solve structure: *SHELXTL* (Sheldrick, 2008 ▶); program(s) used to refine structure: *SHELXL97* (Sheldrick, 2008 ▶); molecular graphics: *SHELXTL*; software used to prepare material for publication: *SHELXL97* and *PLATON* (Spek, 2009 ▶).

## Supplementary Material

Crystal structure: contains datablocks I, global. DOI: 10.1107/S1600536809043451/im2150sup1.cif

Structure factors: contains datablocks I. DOI: 10.1107/S1600536809043451/im2150Isup2.hkl

Additional supplementary materials:  crystallographic information; 3D view; checkCIF report

## Figures and Tables

**Table 1:** Hydrogen-bond geometry (Å, °)

*D*—H⋯*A*	*D*—H	H⋯*A*	*D*⋯*A*	*D*—H⋯*A*
C3—H2⋯O2^i^	0.93	2.35	3.205 (3)	153
C6—H4⋯N1^ii^	0.96	2.62	3.582 (3)	177

Symmetry codes: (i) 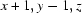; (ii) 
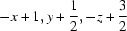
.

**Fig. 1.**
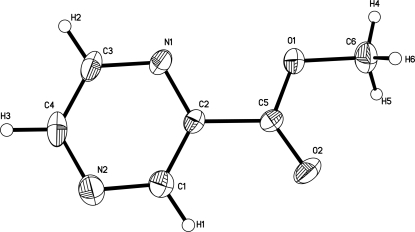
The molecular structure of (I), with atom labels and 30% probability displacement ellipsoids for non-H atoms.

**Fig. 2.**
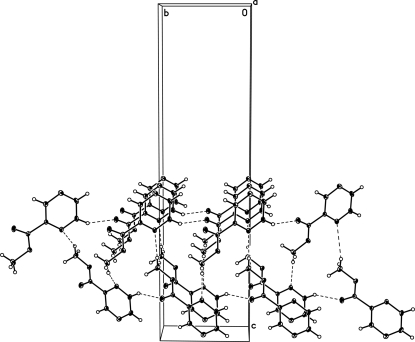
The packing of (I), viewed down the a axis, showing one layer of molecules connected by C—H···O and C—H···N hydrogen bonds (dashed lines).

## supplementary crystallographic information

### Comment

Heteroaromatic esters are more easily reduced than the corresponding free acids (Boechat *et al.* 2005). The title compound, (I) (Fig. 1), [C_6_H_6_N_2_O_2_], was obtained as an intermediate in the synthesis of another pyrazine-based compound.

All non-hydrogen atoms of (I) are coplanar. The maximum deviation from the mean plane is 0.1249 (4) Å for O2 and the mean deviation is only 0.0488 (3) Å. The almost perfect planarity of the molecule reflects its efficient π-conjugation.

There are no classical hydrogen bonds present in the crystal structure (Spek, 2009). Nevertheless, there are weak C—H···O and C—H···N hydrogen bonds (Table 1, Desiraju & Steiner, 1999) linking the molecules into an infinite three-dimensional network [Fig. 2].

### Experimental

Compound (I) was prepared following a procedure published by Kim *et al.* (2004), but the product is not "pale brown" but colorless. Elemental analysis Calcd: C 52.17, H 4.38, N 20.28%. Found: C 51.87, H 4.02, N 20.14%.

### Refinement

Since the compound itself is achiral and in the absence of significant anomalous dispersion effects, Friedel pairs were averaged. All H atoms were fixed geometrically and allowed to ride on their parent atoms, with C—H distances in the range 0.93–0.97 Å, and with *U*_iso_(H) = 1.2 *U*_eq_(C) for CH groups of the pyrazine ring and *U*_iso_(H) = 1.5 *U*_eq_(C) for the methyl group.

### Crystal data

**Table:**

C_6_H_6_N_2_O_2_	*F*(000) = 288
*M**_r_* = 138.13	*D*_x_ = 1.424 Mg m^−^^3^
Orthorhombic, *P*2_1_2_1_2_1_	Mo *K*α radiation, λ = 0.71073 Å
Hall symbol: P 2ac 2ab	Cell parameters from 378 reflections
*a* = 3.865 (2) Å	θ = 1.6–25.5°
*b* = 6.690 (4) Å	µ = 0.11 mm^−^^1^
*c* = 24.92 (2) Å	*T* = 298 K
*V* = 644.4 (7) Å^3^	Needle, colourless
*Z* = 4	0.32 × 0.12 × 0.05 mm

### Data collection

**Table:**

Bruker SMART CCD area-detector diffractometer	757 independent reflections
Radiation source: fine-focus sealed tube	505 reflections with *I* > 2σ(*I*)
graphite	*R*_int_ = 0.080
π and ω scans	θ_max_ = 25.5°, θ_min_ = 1.6°
Absorption correction: multi-scan (*SADABS*; Bruker, 2000)	*h* = −4→4
*T*_min_ = 0.980, *T*_max_ = 0.994	*k* = −7→8
3378 measured reflections	*l* = −30→22

### Refinement

**Table:**

Refinement on *F*^2^	Primary atom site location: structure-invariant direct methods
Least-squares matrix: full	Secondary atom site location: difference Fourier map
*R*[*F*^2^ > 2σ(*F*^2^)] = 0.066	Hydrogen site location: inferred from neighbouring sites
*wR*(*F*^2^) = 0.153	H-atom parameters constrained
*S* = 1.05	*w* = 1/[σ^2^(*F*_o_^2^) + (0.0698*P*)^2^] where *P* = (*F*_o_^2^ + 2*F*_c_^2^)/3
757 reflections	(Δ/σ)_max_ = 0.001
91 parameters	Δρ_max_ = 0.18 e Å^−^^3^
0 restraints	Δρ_min_ = −0.17 e Å^−^^3^

### Special details

**Table:**

Geometry. All e.s.d.'s (except the e.s.d. in the dihedral angle between two l.s. planes) are estimated using the full covariance matrix. The cell e.s.d.'s are taken into account individually in the estimation of e.s.d.'s in distances, angles and torsion angles; correlations between e.s.d.'s in cell parameters are only used when they are defined by crystal symmetry. An approximate (isotropic) treatment of cell e.s.d.'s is used for estimating e.s.d.'s involving l.s. planes.
Refinement. Refinement of *F*^2^ against ALL reflections. The weighted *R*-factor *wR* and goodness of fit *S* are based on *F*^2^, conventional *R*-factors *R* are based on *F*, with *F* set to zero for negative *F*^2^. The threshold expression of *F*^2^ > σ(*F*^2^) is used only for calculating *R*-factors(gt) *etc*. and is not relevant to the choice of reflections for refinement. *R*-factors based on *F*^2^ are statistically about twice as large as those based on *F*, and *R*- factors based on ALL data will be even larger.

### Fractional atomic coordinates and isotropic or equivalent isotropic displacement parameters (Å^2^)

**Table:**

	*x*	*y*	*z*	*U*_iso_*/*U*_eq_	
C1	0.2013 (5)	0.6623 (3)	0.93604 (7)	0.0546 (6)	
H1	0.0959	0.7714	0.9524	0.065*	
C2	0.2602 (4)	0.6690 (2)	0.88246 (6)	0.0371 (5)	
C3	0.5035 (6)	0.3674 (2)	0.88599 (7)	0.0561 (6)	
H2	0.6133	0.2594	0.8698	0.067*	
C4	0.4367 (6)	0.3607 (3)	0.93986 (7)	0.0604 (6)	
H3	0.4999	0.2470	0.9589	0.072*	
C5	0.1459 (4)	0.8493 (2)	0.85163 (6)	0.0395 (5)	
C6	0.1405 (5)	1.0165 (2)	0.76991 (8)	0.0642 (7)	
H4	0.2569	1.0139	0.7359	0.096*	
H5	0.1955	1.1385	0.7883	0.096*	
H6	−0.1049	1.0089	0.7643	0.096*	
N1	0.4157 (4)	0.52391 (19)	0.85628 (6)	0.0479 (5)	
N2	0.2869 (5)	0.5082 (2)	0.96584 (6)	0.0665 (6)	
O1	0.2516 (3)	0.84861 (17)	0.80180 (4)	0.0514 (4)	
O2	−0.0298 (4)	0.97530 (17)	0.87108 (5)	0.0690 (5)	

### Atomic displacement parameters (Å^2^)

**Table:**

	*U*^11^	*U*^22^	*U*^33^	*U*^12^	*U*^13^	*U*^23^
C1	0.0681 (13)	0.0448 (11)	0.0508 (10)	0.0017 (11)	0.0019 (11)	0.0020 (10)
C2	0.0335 (8)	0.0284 (8)	0.0494 (10)	0.0006 (8)	0.0029 (9)	0.0011 (8)
C3	0.0621 (12)	0.0330 (9)	0.0733 (12)	0.0112 (11)	−0.0098 (11)	0.0040 (10)
C4	0.0677 (13)	0.0406 (10)	0.0728 (12)	0.0014 (11)	−0.0187 (12)	0.0199 (10)
C5	0.0409 (10)	0.0302 (8)	0.0475 (10)	−0.0001 (9)	0.0019 (9)	0.0023 (9)
C6	0.0719 (15)	0.0541 (11)	0.0666 (13)	0.0086 (12)	−0.0045 (11)	0.0180 (11)
N1	0.0535 (9)	0.0344 (7)	0.0559 (9)	0.0089 (8)	−0.0002 (8)	−0.0017 (8)
N2	0.0891 (12)	0.0549 (10)	0.0554 (10)	0.0024 (11)	−0.0065 (10)	0.0083 (9)
O1	0.0664 (8)	0.0412 (6)	0.0465 (7)	0.0105 (7)	0.0004 (7)	0.0083 (6)
O2	0.0935 (10)	0.0428 (7)	0.0705 (9)	0.0264 (8)	0.0187 (8)	−0.0033 (7)

### Geometric parameters (Å, °)

**Table:**

C1—N2	1.312 (2)	C4—N2	1.315 (3)
C1—C2	1.355 (2)	C4—H3	0.9300
C1—H1	0.9300	C5—O2	1.186 (2)
C2—N1	1.315 (2)	C5—O1	1.307 (2)
C2—C5	1.497 (2)	C6—O1	1.441 (2)
C3—N1	1.327 (2)	C6—H4	0.9600
C3—C4	1.368 (3)	C6—H5	0.9600
C3—H2	0.9300	C6—H6	0.9600
			
N2—C1—C2	122.76 (17)	O2—C5—O1	124.72 (15)
N2—C1—H1	118.6	O2—C5—C2	122.14 (15)
C2—C1—H1	118.6	O1—C5—C2	113.11 (14)
N1—C2—C1	122.77 (15)	O1—C6—H4	109.5
N1—C2—C5	118.36 (15)	O1—C6—H5	109.5
C1—C2—C5	118.87 (15)	H4—C6—H5	109.5
N1—C3—C4	121.68 (17)	O1—C6—H6	109.5
N1—C3—H2	119.2	H4—C6—H6	109.5
C4—C3—H2	119.2	H5—C6—H6	109.5
N2—C4—C3	122.83 (17)	C2—N1—C3	114.99 (15)
N2—C4—H3	118.6	C1—N2—C4	114.94 (16)
C3—C4—H3	118.6	C5—O1—C6	115.33 (14)

### Hydrogen-bond geometry (Å, °)

**Table:**

*D*—H···*A*	*D*—H	H···*A*	*D*···*A*	*D*—H···*A*
C3—H2···O2^i^	0.93	2.35	3.205 (3)	153
C6—H4···N1^ii^	0.96	2.62	3.582 (3)	177

Symmetry codes: (i) *x*+1, *y*−1, *z*; (ii) −*x*+1, *y*+1/2, −*z*+3/2.
